# A defence of identity for persons with disability: Reflections from religion and philosophy versus ancient African culture

**DOI:** 10.4102/ajod.v8i0.490

**Published:** 2019-04-23

**Authors:** Patrick Ojok, Junior B. Musenze

**Affiliations:** 1 Department of Community and Disability Studies, Kyambogo University, Kampala, Uganda

## Abstract

**Background:**

Religion and philosophy follow the Hegelian dialectic, man as thesis, evil as antithesis and ideal man or God the final synthesis, locking out persons with disability stating that they don’t meet the criteria of being human persons. In contrast, persons with disability were accepted in ancient Africa and their disorder was not shown as a physical handicap.

**Objectives:**

The objective of this article was to critically examine how disability is constructed in philosophy and religion in comparison with African culture, in the shaping of disability identity as a form of humanity.

**Method:**

This article undertook a document review of both grey and peer reviewed literature. The papers reviewed were identified and screened for relevance, then analysed with the aim of comparing the portrayal of disability in philosophy, religion and ancient Africa.

**Results:**

Our analysis revealed that African cultures revered the disability identity, as opposed to philosophy and religion that portrayed it as abnormal. A person with disability was accepted in ancient Africa and given a visible role in society suggesting their integration in daily life activities while their disability was believed to be a blessing from the gods.

**Conclusion:**

Religion and philosophy have incredibly alienated persons with disabilities with linguistic and derogative identities. Whereas African spiritualism inherently glorified and/or approved disability, in today’s Africa, persons with disability are increasingly objectified and abused because of ignorance and harsh economic conditions. Nevertheless, the contemporary mistreatment of people with disabilities (PWDs) does not reflect a true African culture but is a symptom and a consequence of the material and economic injustice that PWDs encounter.

**Keywords:**

disability; identity; philosophy; African; religion.

## Introduction

Human identity even in contemporary philosophical logic and metaphysics brims with controversy. Definitions of identity have caused racism, institutionalisation and denial of rights. Most saddening is the linguistic and situational identity atoned to persons with disabilities by two traditional disability models, the ‘social and medical models’, exhibiting great reliance on the utopian image of man that philosophy and religion historically have constructed as a measure for human identity. The medical model views disability as a defect or sickness that must be cured through medical intervention (Kaplan 1999). This perspective assumes that the function of intervention or treatment is to *fix, cure* or *ameliorate* the disability so that the individual will be better able to function in society (Susan 1996). However, such categorisations create social class systems where some humans are seen as less human than others, and as a consequence persons with disability are embroiled in the fracas of identifying with normalcy. The social model creates a social position for an individual that is constructed in response to widely held notions of normalcy, in that ‘disability is the attribution of corporeal difference – not so much a property of bodies, as a product of the cultural rules about what bodies should be or do’ (Garland 1997; Oliver 1990). Byrne (2000) states that persons with disabilities remain philosophically marginalised as such individuals may fail to live up to the strict philosophical standards associated with human nature as rational and able-bodied. Disability Studies continues the task of defining man’s identity that fits contemporary conceptions of human identity.

## Philosophical discussion

Whereas philosophy did not initially matter in Greece and the Roman world, all life experiences of antiquity were guided by myths and legends (Kerenyi 1974). These gave tales about gods, their appearance, occupation and man–god intercessors. Hamilton (1942) in her book *Mythology* described a scene in which Oedipus the king of Thebes consults Tiresias, the old blind prophet of Thebes, to find out who had killed King Laius, as his kingdom was facing tragedy as a consequence of the king’s death. Furthermore, the god Hephaestus, one of the multitude of Greek deities, was himself perceived as lame, although he did not stand as a symbol of disability. He was the god of the forge, a skilled artisan who is said to have created some of the wonders of Greek mythology such as the shield of Achilles (Hard 2004).

It is tempting to believe that, given their contemplative abilities, the ancient Greeks had a similar conception of disability identity based on contemporary terminology; however, it was much different for if they did, there would be so many persons with disability. Martha Rose (2003) in *The Staff of Oedipus* stated that persons with disability in ancient Greece were not a clearly defined subcategory of human beings and it would be archaic to investigate the phenomenon of disability in Greece from the perspective of a contemporary disability model. It was common to account for a lost body part with an increased ability of the use of another sense or bodily feature. For example, a Greek philosopher named Dio Chrysostom, who was banished from Rome by Emperor Domitian, thought that blindness was not unique to Homer, but that all poets should be blind. He said, ‘moreover, all the poets are blind, and they do not believe it is possible for anyone to become a poet otherwise’ (Hartsock 2008).

Indubitably, Plato sits at the foundation of philosophical discourse of the West, and contemporary authors still turn to his writings when attempting to support argumentation about ancient Greek culture. The *Republic*, one of his dialogues, detailed the nature of traditional scholarship and how it rendered people with disability in ancient Greece inconsequential and invisible (Rist 1986). It was one of the first philosophical texts to specifically argue that an ideal city governed by reasonableness should actively kill individuals with intellectual and physical disabilities because such individuals embody injustice as the lack of order (Jowett 1986).

In Book IV of the *Republic*, Socrates argues that:

to produce health is to establish the elements in a body in the natural relation of dominating and being dominated by one another, while to cause disease is to bring it about that one rules or is ruled by the other contrary to nature. (Kromm 2002:3)

Health is thus an objective good associated with order, beauty and proper functionality as a type of harmony, and disease and dysfunction are associated directly with disorder, ugliness, the bad condition of the soul and, most importantly for the Republic, injustice as a type of disharmony (Broad 1953). Socrates’ argumentation pioneered a philosophical conception of disability as a type of deficiency while setting off the Neoplatonic movement that preceded Plato’s Academy.

A Neoplatonist, John Scotus Erigena, theorised that the universal (the ideal) is the essence of reality and that each particular object is contained within the universal and is a product of the universal (Carabine 2000). According to Macfarlane and Roland (2004), Scotus meant that the more universal an object is, the more real it is; the more of humanity a particular person possesses, the more real ‘he or she’ is and problems of material existence such as disease and disability are a consequence of deprivation of a higher good – the complete reality of perfection.

The charity model of disability is traceable to medieval times. The medieval period was a moment for development of beliefs and most explicitly was the development of Christianity. The church particularly refused to ordain any person with disability into ministry. The canon law and theological books accorded persons with disability the title *sinners*. However, with the presence of monasteries and churches in the medieval period, charity was always offered to these groups of people, and in particular Saint Louis granted blind people a rare legal right to beg on the streets of Paris (Wheatley 2002).

In *Being and Essence*, St. Thomas Aquinas, a medieval philosopher using Aristotelian metaphysics, devoted much thought to the question, ‘What does it mean to be?’ And using the process called *hylomorphism* (the doctrine that physical objects result from the combination of matter and form), he specified that for humans, matter is substance and is corporeal, extended and has a desire for form, but the moment is deprived of form; it causes instability (Gilson 1955). Matter first receives universal form, that is, substance and substantial form individuates matter. It receives further perfections from other forms, so that there is a plurality of forms in any given body that make up one universal form, so that if any of the plural form is devoid, then matter defaults in that universal form and leads to disability (O’Daly 1987). In other words, when one lacks a leg, he has deprived his substance of the form of the leg and such a person is a pseudo human.

René Descartes, a modern philosopher, is well known for having ascertained the soul’s existence and doubted bodily knowledge by the statement *cogito, ergo sum*, that is, ‘I think, therefore I am’. He assumed that the soul was in the body akin to a captain piloting a ship, giving the soul the primacy of existence with a mechanical body whose essence was to obey the soul while performing the activities of the soul when in its ‘normal state’. While reflecting on the relationship between the body and soul, Sen (1992) noted that:

the mathematical precision that Descartes’ ‘soul’ would possess would be faulty if a disabled body transmitted empirical knowledge to the soul, since the soul would not have the idea of a normal human body. (pp. 14–17)

In relation to the Darwinian theory of evolution, the story of survival of the fittest indicates that persons with disabilities were an *impure race* who by the act of natural selection was not suitable for existence and competition with persons with no disability. Social Darwinism portrayed evolution as a struggle for existence in which superior races survived and inferior ones perished, which among others included people with disability and non-European races, a fact that the Nazi Germany used to exterminate many persons with disabilities as well as Jews, as they were considered non-human (Galton 1998). Darwinism and the eugenics movement (Paul 2008) provided:

a simple solution to the complex issues of physical disorders, mental illness, developmental disabilities and changing social conditions, by eliminating what the movement supporters considered to be hereditary flaws through selective reproduction at many camps in Germany, where thousands of disabled people and other ‘undesirables’ or ‘useless eaters’ were exterminated during the Nazi regime. (pp. 57–80)

In conclusion, philosophy discourse attempts to use rational and substance principles to derive an intelligible basis for human identity that deny those individuals (e.g. persons with disability) who lie outside of, and beyond, reason in its strict sense. Symmetrical conceptions of disability identity cannot be fully deduced from philosophical principles alone without incompleteness, but only empirical to rational rather than a priori rational considerations can adequately address the individuality and contingency of a concrete disability identity.

## Religious discussion

African societies have been largely inhabited in the realm of the Judeo-Christian religions (i.e. Islam, Christianity and Judaism) and yet we cannot forego the fact that religious beliefs greatly influence perceptions of persons with disabilities, of themselves, others and the world (Ahmad 2015). Religion has its etymology from the Latin word *religare*, which means ‘to tie, to bind’, and spirituality is a relationship between an individual and what excites him or her with which she or he has an emotional connection (Donald 2006). We would say that spirituality and religion are not synonymous with each other: Although spirituality is necessary for religion, religion is not necessary for spirituality.

Religion has huge psychological acceptance, given its use of spirituality and the fear that comes with its negation, and it is very clear that humans are afraid of what they cannot see coming. Just like the philosophers, religions figuratively use disability to foster their views. For example (Ahmad 2015:4), the Qur’an states:

have they, then, never journeyed about the earth, letting their hearts gain wisdom, and causing their ears to hear? Yet, verily, it is not their eyes that have become blind – but blind have become the hearts that are in their breasts! (22:46).

The word ‘blind’ is used here to refer to the loss of spiritual insight and not the loss of vision or eyesight in the physiological sense. Such verses regarding the blind, the deaf and the mute leave us with the conclusion that the words in the Qur’an are intended to signify one who is spiritually or morally bereft and not one who is physically disabled.

In the same manner, the Bible frequently uses disabling language or imagery while discussing topics unrelated to persons with disability. For example, in Isiah 56:10, the prophet declares, ‘Israel’s lookouts are blind, all of them do not know; all of them are mute dogs that are not able to bark; dreaming, lying down, loving to be drowsy’ (Olyan 2008). As Olyan points out, this verse does not discuss the persons with disability themselves; it uses the words ‘blind’ and ‘mute’ as metaphors to criticise the ineffective efforts of Israel’s leadership. Similarly, several other biblical prophesies or curses use disability imagery to describe the moral or ethical conditions of presumably an audience of persons without disability (cf. Dt 28:28–29;^1^ Is 29:9; 59:10^2^).

Such passages may tell us what a particular prophet thought about how the Israelites conducted themselves. They tell us very little, however, about the actual living conditions or everyday experiences of people with disability in ancient Israel. Instead, these passages frequently use language and imagery of disability to describe the experiences and struggles of, presumably, the persons without disability (Dorman 2007).

Judeo-Christianity chooses a certain vulnerable and presumed voiceless part of society (e.g. persons with disability) that then becomes a scapegoat for all tragedy and mischief. They become the basis of explanation of sin, and all atonements that project a lack of faith in those religions. The identity of persons with disability is henceforth created after such undesirable conditions are outlined.

Through faith and prayer, there are allegations of healings where it is said that ‘the blind see and the deaf hear’; hence persons with disability are presumed to go to church only for healing (Boaz 2015). There is however scanty scientific literature on how these people have their bodies restored, if at all; it is not theatrical play and whereas these healings do happen occasionally, a person who does not get healed is usually said to be a seasoned sinner. In fact, Boaz challenges the dominant belief that persons with disabilities need a healing miracle and posits that they go to church to primarily seek counsel, comfort and company.

In conclusion, the implication of religion and philosophy in the use of stigmatising vocabulary, figuratively or imagined, whether used to refer directly to members of stigmatised groups or derivatively to things associated with such groups, exemplifies discrimination and social stigma not so much in how the group in question is being distinguished but in how language and other associations are being used in reference to their identity (Routledge 1998).

## Ancient African discussion

African societies are deeply rooted in spirituality as opposed to religion, but Western-educated African scholars tend to confuse religion with spiritualism, thereby misrepresenting traditional Africans as religious. Mbiti is credited with some of East Africa’s great literary works in the colonial and postcolonial periods, and in one of his major works, *Introduction to African Religion*, he claimed that ‘Africans are notoriously religious people’ (Mbiti 1991), meaning that Africans, like other religious sects, have uniquely scripted forms of worship, but this is not the case. Africans have spiritual systems that coexist within their traditional cultures, which are not religions but emotive connections (Kasomo 2010).

Ancient African spiritual systems that existed were based on the reverence of nature and environment. These systems in antiquity revered the object of humans, equating humans with the status of a deity. For example, most archaeological artefacts in Egypt and Sudan on temple walls depicted humans as divine and their bodies as holy, including the body that was said to be disabled (Kozma 2006). Western anthropologists did not understand African spiritual hermeneutics and equated it to evil while labelling it as animism because they came from backgrounds of scripted religions, which was not the case with Africa.

The magnificent past of Egypt, recorded with iconic art and preserved on many temple walls, tombs and artefacts, witnesses to ancient Egypt as the convergence of the multicultural systems of Africa (Trigger 1993). Many artistic impressions, generally realistic, opted to explain the real situation of Africans. The triangular shape of the tombs, most being pyramids, were all over Africa. It is most likely that the present burial of royals, nobles and distinguished personalities in pyramid-like structures even currently in Uganda is an affirmation of the African experience in a smaller Egypt (Lucas & Haris 1999).

According to Smith (1949), ancient Egyptian art in general adopted certain rules and principles, among which was the representation of the kings and tomb owners in an idealistic body in certain postures and situations. This was not the case when dealing with minor figures. Minor figures were represented in various postures, performing different jobs. This practice was probably responsible for depicting some of these figures with actual disabilities and deformities in ways that expressed cultural and social acceptance of people with disability in general.

African spiritualism inherently glorified and/or approved disability (Lawal 2005). In ancient Kemit, physical disabilities or body deformities were considered as divine spiritual attributes granted to humans by the gods. This was expressed in representing certain gods with misshapen bodies or as dwarfs, like the gods Bes, Hapi, forms of Ptah and Ptah-Sokar-Osiris (Ebeid 1999). Ancient Egypt accommodated disability and these groups of people always made up households of the kings and high officials. Some of those persons with disability attained high positions in the ancient Egyptian courts, for example the dwarfs Seneb and Khnumhotep, as well as Roma, the doorkeeper who had a shortened leg (Cody 2004).

In ancient Africa, the artistic sources provide a rich legacy and documentation of individuals with physical disabilites positions and engagement in the context of daily life activities in ancient Egypt. All classes of people with physical disability were likely accepted in ancient Egypt and were given a visible role in society (Weeks 1970). Moreover their disorder was not shown as a physical handicap but a blessing from the gods. Several high-ranking dwarfs, especially from the Old Kingdom, achieved important status and had lavish burial places close to the royal cemetery. Their costly tombs and statutes carved with hieroglyphs indicate their high-ranking position (Smith 1949).

The acceptance of disability is reflected in myths from other parts of Africa as well. The Yorùbá population of West and Central Africa narrate that *Obatala* [God the creator], tired of just his cat as a companion, decided to create humans to share the Earth with him. Working tirelessly, he moulded figures of men and women shaped like himself. Eventually, he grew exhausted and drank some palm wine to refresh himself, which got him drunk, and he was unable to model the clay properly, resulting in the creation of persons with disability (e.g. dwarfs and albinos) (Ford 2000).

Waking up from his drunk state, he saw the malformed beings. Filled with compassion and remorse, he swore never again to drink palm wine and would be the protector of those who have been created with deformities and imperfections. (p. 16)

According to Wole Soyinka, a Nobel laureate and a Yorùbá, this story brings the god firmly within the human attribute of fallibility:

… since human fallibility is known to entail certain disharmonious consequences for society, it also requires a search for remedial activities, and it is this cycle which ensures the constant regenerative process of the universe (Ford 2000, p. 16).

By bringing the gods within this cycle, a continuity of cosmic regulation is guaranteed in Africa (Linton 1998).

To support the argument that (ancient) African beliefs about disability are not always negative (e.g. the representation of certain Kemit gods as disabled), the Yorùbá folklore similarly identifies a limping trickster god (Èshù), whose impairment gives him access to multiple realities of both the natural world and that of the gods. The Yorùbá Èshù therefore represents a character with disability or god who provides superior insights into the phenomenal or supernatural world (Quayson 2012). This example of the powers of Èshù fits Quayson’s categorisation of the representation of disability as ritual insight.

In contemporary Africa, persons with disability are being hunted for magical potions, discriminated against and their accessibility in society made difficult (Tanner 2010). Nonetheless this has not stopped the Vadoma people of Zimbabwe – also known as the ‘ostrich people’, who have a rare condition of their feet – from perpetuating a positive spiritual symbolism related to persons with disabilities. According to Farrell (1984), these people have a condition known as ‘ectrodactyly’ in which the middle three toes are absent and the two outer ones are turned in. This condition makes it difficult for them to run or even wear conventional shoes. However, the condition is regarded with pride among the Vadoma people, and they forbid members to marry outside the group. The story of the Vadoma people is a vivid example that affirms ancient African sentiments about disability amidst the change that we now see.

In contrast, in today’s Africa, persons with disability are increasingly becoming objects of pity and abuse because of ignorance and harsh economic conditions. Evidence of denying the identity of persons with disability abounds in churches where pastors exercise miraculous healing (Boaz 2015). As well, certain persons with disabilities, particularly albinos, are being killed because of the false belief that burying their heads in the foundation of a building makes the house owner rich. In Uganda, a local newspaper carried a story of the gruesome murder of two children with disabilities by their own mothers. The mothers allegedly cited strong negative stigma and being divorced for giving birth to ‘abnormal children’ as the (inexcusable) reasons for killing their own babies (Gerald & Luk 2018).

## Conclusion

We have attempted to show that ancient African cultures revered the disability identity, unlike philosophy and Western religion that portray it as cursed or abnormal. Philosophy and religion have created enough damage for persons with disability by creating imagery and figurative compositions to alienate them from humanity. Artistic sources provide a rich legacy and documentation of the positions of persons with disability in daily life in ancient Africa and in particular Egypt. A person with disability was accepted in ancient Africa and given a visible role in the society, witnessed in present time among the Vadoma people. Furthermore, their daily activities suggested integration in daily life and their disorder was not shown as a physical handicap but a blessing from the gods; no one worried over a disability as the identity of all was of humanity. And yet in the contemporary situation, religion and philosophy have done much to alienate persons with disabilities with linguistic and situational derogative identities. The modern disavowal of persons with disability does not reflect the true African culture but is symptomatic and a consequence of the material hardships and economic injustices that persons with disability face.

## Footnotes

1 The Lord shall smite thee with madness, and blindness, and astonishment of heart: And thou shalt grope at noonday, as the blind gropeth in darkness, and thou shalt not prosper in thy ways: and thou shalt be only oppressed and spoiled evermore, and no man shall save thee.

2 We grope for the wall like the blind, and we grope as if we had no eyes: we stumble at noon day as in the night; we are in desolate places as dead men.
